# Experiences of Life and Intersectionality of Transgender Refugees Living in Italy: A Qualitative Approach

**DOI:** 10.3390/ijerph182312385

**Published:** 2021-11-25

**Authors:** Fau Rosati, Valentina Coletta, Jessica Pistella, Cristiano Scandurra, Fiorenzo Laghi, Roberto Baiocco

**Affiliations:** 1 Department of Social and Developmental Psychology, Faculty of Medicine and Psychology, Sapienza University of Rome, 00185 Rome, Italy; coletta.1014936@studenti.uniroma1.it (V.C.); jessica.pistella@uniroma1.it (J.P.); fiorenzo.laghi@uniroma1.it (F.L.); roberto.baiocco@uniroma1.it (R.B.); 2 Department of Neuroscience, Reproductive Sciences and Dentistry, University of Naples Federico II, 80131 Naples, Italy; cristiano.scandurra@unina.it

**Keywords:** transgender, refugee, minority stress, trauma, intersectionality, ethnicity, gender, transfeminist, religious coping, gender affirmation

## Abstract

Transgender refugees are at risk of experiencing increased minority stress due to experiences of trauma in their country of origin, and the intersection of multiple marginalized identities in their host country. Adopting a transfeminist and decolonial approach, the present study aimed at exploring transgender refugees’ experiences of life and migration. A semi-structured interview protocol was developed, grounded in the perspectives of minority stress and intersectionality. Participants were five transgender refugees (four women and one non-binary) from different cultural/geographic contexts, professing different religions. Using thematic analysis, the researchers identified three themes: pre- and post-migration minority stress and transphobia; religion as a protective factor for gender affirmation; and individuation and the synthesis of social identities. Participants reported traumatic experiences and the inability to openly live out their gender identity in their country of origin as the main push factors to migration. They also reported feelings of isolation and experiences of victimization during interactions with the Italian asylum services, due to a lack of adequate training, racial prejudice, and transphobia. Participants demonstrated positive individuation, linked to gender affirmation treatments and religious protective factors. The interview protocol may be used by social operators to support the claims of transgender asylum seekers, and to clinically assess transgender people with an immigrant background.

## 1. Introduction

Transgender is an umbrella term that describes many different identity experiences. Transgender people may move away from the sex that they were assigned at birth because they feel that they belong to another—often considered the “opposite”—gender (i.e., transwomen and transmen), or they feel that they belong to both male and female genders, more than these two genders, a fluid gender identity, or no gender (e.g., non-binary, genderqueer, genderfluid, agender) [1,2,3]. Due to the strict gender norms that prevail in most cultures and societies, transgender people face high levels of stigma and discrimination within a wide range of meaningful contexts, including the family, the workplace, physical and mental health care providers, social services, and the broader society [4,5,6]. For gender and/or sexual minorities (e.g., lesbian, gay, bisexual, transgender, queer, and intersex (LGBTQI+) individuals), the sources of stigma and discrimination are both unique, and thereby additive to the general stressors experienced by most people, and chronic—since they depend on relatively stable unequal social and cultural structures. For this reason, LGBTQI+ individuals are understood to suffer from minority stress [7,8].

LGBTQI+ people with multiple marginalized identities [9,10]—including transgender refugees (i.e., people who have been forced to leave their country to escape war, persecution, or stigma due to their gender identity)—may experience increased minority stress, linked to two factors: (1) experiences of trauma in their country of origin [11,12] and (2) the intersection of at least two minority identities (related to gender and ethnicity) in the host country [13,14]. Transgender refugees are often forced to leave their country due to persecution related to their gender identity and expression [15]. Previous studies [11,12,16,17] on LGBTQI+ refugees have found that most report severe and prolonged experiences of trauma prior to migration, including “psychological abuse, physical and sexual assault, corrective rape, forced conversion therapy, blackmail, and public shaming” [12] (p. 14).

In addition to the trauma that many transgender refugees experience in their country of origin, they may also experience secondary victimization in their host country, due to the lack of preparedness of both institutional actors involved in international reception and protection systems and the bureaucratic system in which the international protection device is based [14,18]. The standards and mechanisms of international protection published by the Council of Europe include “sexual orientation and gender identity” (SOGI) guidelines linked to international human rights. Specifically, SOGI guidelines extend the right of asylum to LGBTQI+ people who have fled their country due to persecution or victimization based on their sexual orientation or gender identity; thus, LGBTQI+ migrants have a greater likelihood of obtaining refugee status. At the same time, the SOGI mechanism is based on “valid” motivations linked to sexual orientation and gender identity, and this criterion may be used to justify invasive and at times accusatory investigations into asylum seekers’ lives, including their trauma and intimate experiences [19]. In this process, LGBTQI+ asylum seekers must prove their non-heterosexual orientation or non-conforming gender identity, embodying and identifying with a clear identity category, as conceived by Western culture, rather than their own.

Furthermore, in relation to cisgender and heterosexual refugees, transgender refugees experience additional risk factors: (1) they may be rejected from their communities due to their gender identity [17]; (2) they may hesitate to turn to LGBTQI+ groups in the host country for various reasons, including not recognizing themselves in Western gender/sexual categories or fears of further discrimination based on ethnicity [20]; (3) they generally show poorer health outcomes compared to the general population, and other minority groups that do not experience the intersection of multiple oppressions (e.g., native LGBTQI+ people or cisgender/heterosexual refugees) [15,21]; and (4) they generally have a lower socioeconomic status, due to fewer employment opportunities (this, in turn, may push them to engage in sex work when they would not otherwise choose to do so) [22,23].

The intersectional perspective can be extremely useful in investigating how multiple social identities (e.g., gender, ethnicity, immigrant background, religion, social class) interact to delineate different axes of oppression and privilege, influencing life opportunities and conditions [24]. This perspective is based on a conception of race/ethnicity and gender as structural categories—rather than individual characteristics—defined by culture and society [25]. Therefore, it considers how social identities relate to the power dynamics rooted in social contexts and institutions, creating unique experiences of inequality.

Despite the growing interest in immigration issues demonstrated by various scientific disciplines (including psychology), there have been few empirical studies conducted on transgender refugees in Europe [18,26]. Previous research on sexual minority immigration has typically drawn on samples combining both non-heterosexual and transgender participants, thereby potentially overlooking the specific challenges faced by transgender subjects [9,14,17,18,27,28]. Yet, transgender refugees represent a population deserving specific attention, as they are more likely to experience violence, sexual assault, and being involved in human trafficking when compared to other minority groups (e.g., cisgender migrants). At the same time, they risk being excluded from anti-trafficking protection, due to a lack of institutional knowledge and competence on the topic of transgender identities [23].

In Italy, where the present research was conducted, studies addressing this topic are lacking, and there are no statistical data on the migration of transgender people, aside from limited data on the emerging phenomenon of human trafficking and sex work exploitation involving transgender populations from Brazil, Colombia, and Thailand [22]. The Italian state agency in charge of integration projects for refugees defines the achievement of socioeconomic autonomy as a primary objective [29]. Transgender refugees are considered a vulnerable population, since they are more likely to be involved in human trafficking and to experience psychological, physical, and sexual violence, with severe consequences for physical and mental health. Thus, once they have obtained international protection, they should benefit from easier access to work and social health care. However, recent research has shown a significant lack of institutional support for individuals who have been granted refugee status, in terms of psychological and economic assistance [30]. To date, most of the support and assistance for transgender asylum seekers and refugees has been administered by grassroot organizations [31].

The present study aimed at enriching the empirical literature and providing a useful instrument to gather information on the topic of the forced migration of the transgender population. The overarching goal of the study was to collect preliminary data on transgender refugees’ experiences of pre- and post-migration and their intersecting ethnic and gender identities, using a semi-structured interview grounded in minority stress and intersectionality perspectives [32,33]. Specifically, the study aimed at investigating: (1) experiences related to the pre- and post-migratory journey; (2) the intersectionality of multiple minority identities, such as ethnic and gender identities; and (3) risk and protective factors related to migration and intersectionality.

## 2. Materials and Methods

### 2.1. Participants and Procedures

The sample was comprised of five transgender refugees, who were recruited through the LGBTQI+ Migrant Office of an important Italian grassroots organization engaged in the health of Italian and migrant transgender individuals. All participants spoke good Italian, though with different levels of fluency (range of stay in Italy: 3–18 years). All interviews were conducted in Italian. Participants included four transgender women and one non-binary transgender intersex person, aged 26–47 years (M = 31.6; SD = 4.18).

The five participants had migrated from Cuba, Brazil, Pakistan, Libya, and Turkey, respectively. With regards to religion, one participant was Buddhist, one was Muslim, and three were Christian of different sects (i.e., Pentecostal, Catholic, and Protestant, respectively). Concerning level of education, two participants had a middle school diploma, one had a high school diploma, and two had a bachelor’s degree. All participants described their socioeconomic status as low/very low, and all but one were unemployed. Table 1 presents detailed information for each participant.

The interview was developed by the research team, which was also composed of transgender and non-heterosexual researchers—some of whom had directly engaged in the Italian reception system for transgender asylum applicants, holding protection status. All research members were Italian and White, thus not sharing the racial/ethnic minority status of the participants. This different positioning was carefully considered to acknowledge and reduce bias, prejudice, and unequal power dynamics between the researchers and the participants, using a transfeminist [34] and decolonial [35] methodological approach. The lead interviewer carefully considered the impact of their role, as a White Italian researcher who is asking to non-White migrant participants to share their intimate, traumatic, and painful experiences with them. Moreover, the lead researchers were aware that transgender asylum seekers and refugees are often influenced by the expectations of having to tell the right story to obtain international protection status. This may strongly influence their relationship with any interviewer and the content of their narratives. In the case of this study, the lead interviewer already had a trusted relationship with participants, thus allowing access to genuine stories. Finally, the lead researchers shared with participants important experiences related to transgender identity, which helped to create a safe context during interviews, and embodied critical knowledge and deep understanding during qualitative data analysis.

During the interview, participants were informed about the aims of the study. The informed consent predominantly consisted of stressing the importance of honest answering, participants’ right to leave the interview at any time, and the anonymization of participants’ identities. All participants consented to the reporting of their anonymized narratives in scientific publications. The study was designed in respect of the principles of the Declaration of Helsinki on Ethical Principles for Medical Research Involving Human Subjects, and data were collected in accordance with the General Data Protection Regulation 679/2016. In this paper, pseudonyms are used to ensure the anonymity of all participants. The protocols for conducting the research were approved by the Ethics Commission of the Department of Social and Developmental Psychology, Sapienza University of Rome (protocol number: 744; date of approval: 18 June 2018).

### 2.2. Interview Description

Empirical research has shown that qualitative investigative methods are superior to quantitative methods in their ability to capture the complexity of intersectionality, since they analyze social identities in their interaction, rather than considering them isolated constructs. To this end, we developed a semi-structured interview called “Intersections-T”, based on qualitative instruments used in previous studies of minority stress at the intersection of various forms of oppression and stigmatized identities [32,33]. The interview protocol comprised two main sections: the first pertained to an interactive activity, and the second included content-oriented questions.

#### 2.2.1. Identity Mapping

At the beginning of the assessment, participants were asked to select and define the social identities that they perceived as significant in their life, choosing from paper circles representing six options (sex/gender, sexual orientation, ethnicity, religion, education, and socioeconomic status) and open options, through which they could contribute their own relevant identities beyond the options provided. Each option had three further dimension options (i.e., sizes of circle: small, medium, and large) that participants could select to indicate the relevance of each social identity, as personally experienced. Participants placed the selected circles on a white sheet of paper, determining the proximity between circles, according to the extent to which they perceived the respective identities as they related to one another.

The instructions for this part were: *“Now we will play a game. As you can see, there are various identities or categories you may feel you belong to somehow. You may also choose other identities you feel are important to you. Each identity is written within these circles. As you can see, the circles are of three sizes: small, medium, and large. You should first choose the identities you feel are important to you and then the size, based on their importance in your life. Place the identities anywhere on the sheet and put those that you deem closest at a shorter distance from each other”*. Participants performed this task twice, with reference to two periods: the present moment (i.e., post-migration) and the period prior to their migration to Italy (i.e., pre-migration).

With reference to participants’ identity maps, we then asked participants a set of questions concerning their identity construction, and the intersections of their chosen social identities, with a specific focus on the intersections of ethnicity, religion, and gender. Example questions were: *“I would like to understand how, in your opinion, these two identities (gender and ethnicity) relate to each other”; “Tell me about your experience as a [gender label] in the [ethnic community]”;* and *“Tell me about your experience as a [ethnic label] in the Italian LGBTQI+ community”*.

#### 2.2.2. Migratory Experience

The second section of the interview protocol allowed us to explore participants’ life experiences before, during, and after migration. Specifically, a list of questions investigated participants’ motivations to migrate, as well as their challenges, stressors, coping strategies, and resilience (e.g., *“What were the reasons that prompted you to come to Italy? Was your gender identity one of them?”; “Looking back in time, what was the most difficult thing you faced in moving to Italy?”;* and *“What factors helped you manage the challenges you faced?”*). Other questions investigated participants’ potential experiences of discrimination and victimization, both in their country of origin, and in Italy (e.g., *“Would you like to tell me about the problems in your life related to being [gender label] in [country of origin/Italy]? Were there times when you were treated differently for being [gender label]?”*). Finally, more open-ended questions explored participants’ positive perceptions of their life and identity (e.g., *“What is the best thing in your life? What are the things that make you most proud of yourself?”; “Are there any positive aspects of being [gender and ethnic labels] in Italy?”*).

### 2.3. Data Analysis

The interviews were transcribed verbatim and analyzed using thematic analysis [36]. The thematic analysis procedure involved several steps of coding and defining the emerging themes, through comparison between study authors. Specifically, in the first step, the first and second authors (i.e., the lead researchers) independently extracted a series of codes from the transcripts, and discussed the emerging themes in two meetings. In the second step of the analysis, the other research team members conceptualized a final thematic structure, by discussing the experience and knowledge of the lead interviewer and the presentation of emerging codes. Once the thematic structures were defined, the third and final step involved the creation of a table of themes, sub-themes, and related quotations from the interview transcripts.

## 3. Results

### 3.1. Identity Mapping

**Figure 1:**
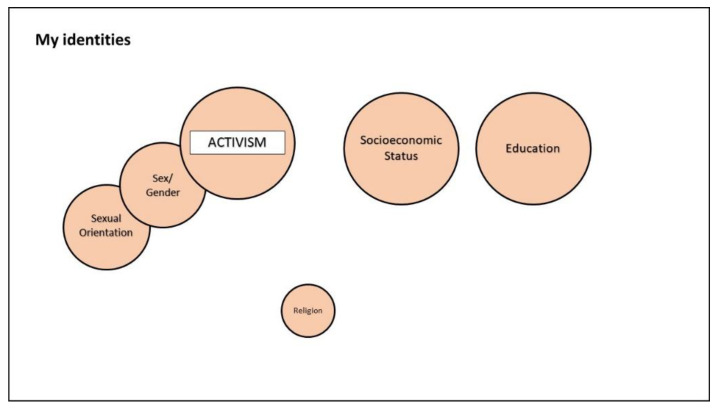
Example of the identity mapping of one participant, referring to the post-migration period.

**Figure 2:**
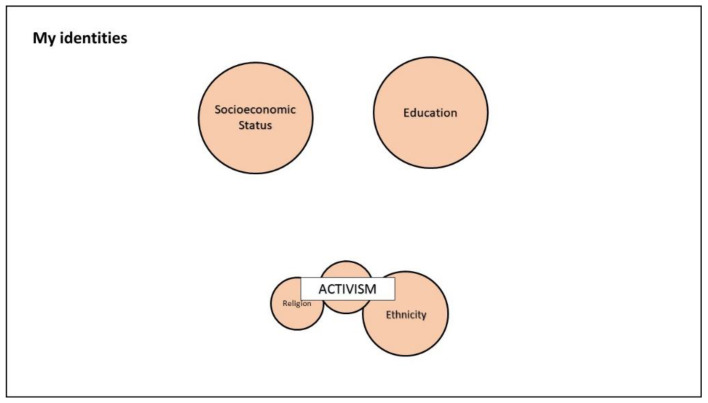
Example of the identity mapping of the same participant, referring to the pre-migration period.

Participants first commented on the social identities that they deemed significant, and the ways in which such identities intersected. Figure 1 and Figure 2 show the identity map of Serra, a 28-year-old Turkish transwoman.

Figure 1 refers to the post-migration period, displaying the social identities that Serra perceived as significant at the moment of the interview. Here, we report an extract from Serra’s comment on the identity mapping exercise, concerning the post-migration period:

[The social identities are] connected because I can’t separate these things: sexual orientation, sex/gender, and activism, although there are other things that are more important to me now. These are important because I don’t feel good, in the sense that being trans, being trans migrant, trans refugee, is not easy and I would like to change my life. For these reasons, I put [them as] connected. [The connection is] not too big. [It’s] medium, because it’s important to me. Why not big if it’s important to me? Ok, they don’t all have to be like me, I’m trans, I’m more heterosexual, trans, woman, but to do activism, to do a good thing for the community, you don’t have to be like me, you don’t have to be a trans woman or trans man. Yes, this is my idea.

Figure 2 refers to the pre-migration period, reporting the meaningful identities that Serra perceived when living in her country of origin. The below quotation from Serra refers to her identity map for this period:

I put socioeconomic status and education [as] both big and above other things […] because the socioeconomic status also in Turkey is important—if not more important than here in Italy—because in Turkey the difference between rich and poor is big. Poor is big, very big. If you don’t have money, you’re… you have no voice, nothing. In my opinion, education in Turkey is more important than here in Italy because in Turkey people are illiterate. […] People are illiterate but not literally. For example, my uncle is a medical professor but he thinks that being LGBT is a psychiatric problem. An example, so I, for example, as a non-Muslim, non-heterosexual, non-cisgender person, I always have to be like this, I always have to be more educated than them otherwise you are always submissive like under the stone so they don’t see you, eh, so these two [are] too important in Turkey and I didn’t put sex/gender, sexual orientation because in Turkey this doesn’t mean anything. For them even being an activist is important to change something but if you don’t live you can’t do activism and I want to live I don’t want to die. In Turkey to do activism for the trans community is too difficult and I was fired because I went [to] pride. And many times it happened many other things, so ok, well, activism for LGBT in Turkey is difficult. Someone has to do this activism but this person to do it is not me, another person can do it but not me because I want to live, to be alive is more important than being an activist in Turkey, in the sense, in this sense. And I put religion still small and medium ethnic in Turkey, linked to activism because in Turkey I belonged to a minority because I was Christian. In Turkey there are not so many Christian or Jewish people, etc., 95% and more are Muslims and to do religious activism is not too dangerous, in Turkey is not too dangerous.

This paper does not seek to analyze the identity mapping section of the interview protocol in depth. However, some extracts reported in the descriptions of the themes were derived from—and in some way facilitated by—this activity. More details regarding the identity mapping exercise are available from the first author, upon request.

### 3.2. Themes

Three main themes emerged from the thematic analysis: (1) pre- and post-migration minority stress and transphobia; (2) religion as a protective factor for gender affirmation; and (3) individuation and synthesis of social identities. Table 2 include the thematic structure and additional representative quotations.

**Content warning:** The following quotations concern episodes of physical and verbal violence perpetrated by families and institutions, as well as other stressful experiences related to transgender and/or refugee identities, which readers may perceive as disturbing. We issue this warning to allow readers to emotionally prepare for the content, or decide to forgo interacting with the material in this section.

#### 3.2.1. Pre- and Post-Migration Minority Stress and Transphobia

Regarding the first theme, it emerged that participants had encountered a range of stressors and traumas throughout their lives—in their country of origin, during their migration journey, and in the host country [12]. Ayoub (29-year-old non-binary transgender person of Libyan origin and Buddhist religion with an Islamic background) recounted the physical and psychological violence that they had experienced since childhood within their family, aimed at “correcting” their gender identity. They defined this as “domestic torture”:

From my father […] it was verbal violence, torture, I experienced domestic torture from my father. I have experienced domestic torture by my father and also my brothers, and they have always tried [to “correct” my gender identity] since I was a child because I was very much towards the male gender. They have tried in every way to feminize my body and my behavior and let’s say the… that violence, I call it violence, it was what I said about to take the hormone, the pill, they tie me up…. my father with the belt puts my hands behind my back with the belt, and they forced me to take… my mother opens my mouth, and they forced me to take the hormone and then other episodes like always threaten to make me marry a man, physical violence in the sense that they beat me and the society beat me… there is indeed, a thing maybe never said there is they tried to make the other person insult me.

Other participants reported the challenges that they experienced during the migratory journey, particularly as transgender women in a predominantly cisgender male group, leading to sleep and livelihood deprivation. Amina (20-year-old transwoman of Pakistani origin and Islamic religion) stated:

During the trip, I slept in the forests. I didn’t eat, I had a fever, I couldn’t walk, I couldn’t climb the mountains, my knee hurt. So, everything is not just one thing, the whole trip was difficult, but I didn’t lose my courage, and I wanted to go to a country where I could be free.

Once in Italy, participants reported experiences of loneliness and institutional transphobia within reception circuits [19,37]. Brenda (47-year-old trans woman of Brazilian origin and Roman Catholic religion) expressed the following sentiment:

The hardest thing when I was alone, I missed everything, there was no one near me who could help me.

Eres et al. [38] associated loneliness with mental health and physical wellbeing in the LGBTQI+ population. Added to this, socially vulnerable groups, such as the elderly, migrants, and asylum seekers, are more susceptible to the effects of loneliness.

Ayoub reported two episodes of discrimination: one at their local Provincial Police Bureau and the other at a Provincial Centre for Adult Education:

So I’ve experienced it a lot in the institutional office–let’s say official–in the police station, in the hospital, in the school, in the municipality, in the post office, everywhere–in the revenue agency, of course. […] Everything [happened]. When you present yourself with your passport with a feminine personality and appear masculine, they ask you: ‘So you want to be a woman? Are you male and want to become a woman? Ah ok you used to be a woman, so people like you exist too. Why did you change your mind when you arrived in Italy?’ […] Then I felt anger, I felt hurt, I felt maybe the first time that I feel equal to an Italian trans citizen because there is all trans people even if Italian citizens–the same things and… basically I was angry, I said I want to talk to the school manager, ’Don’t you dare to say something like that in front of…’, I started to shout. However, he didn’t do anything to me, they didn’t do anything to me. I didn’t take my right anyone defended me not… not even those of the school manager, he was very vulgar that person and indeed he looked at me bad all the school year.

#### 3.2.2. Religion as a Protective Factor for Gender Affirmation

Following Deutsch et al. [39], religion forms the basis for many people’s self-perception and worldview. In the context of the social psychology of religion, Pargament et al. [40,41] defined religious coping as an ongoing process through which individuals attempt to understand and cope with life’s personal and situational demands, using religious thoughts and practices. In the present study, religiosity emerged as an essential area of life for each participant, promoted by a progressive and affirmative religious community and/or a positive personal synthesis between participants’ gender identity and religious tradition [9,16,42].

For example, Maria (34-year-old transwoman of Cuban origin and Evangelical Pentecostal religion) experienced her gender affirmation path as a reparation and returning to “God’s way”:

Actually I didn’t have a bad experience because I was lucky that the church I was attending and the leader of the church were saying that God welcomes everyone as we are and the same God changes you and you have to reshape yourself in God’s way. That’s why we had this problem.

Remarkably, even participants who lacked affirmative religious experiences were able to use religion as a coping strategy, by developing theories about their gender experiences that diverged from traditional theology and religious frameworks, but allowed them to live out their spirituality in a positive way. An example of divergent thinking about gender relative to the dominant Islamic understanding in Pakistan was provided by Amina, who managed to reconcile her Muslim identity with her gender:

Yes, in sura, and it is written, it talks about people, God said I can do what I want, I can give him a son as a daughter, and a daughter as a son. These mean he is talking about transgender people. A son is born becomes a daughter. So he talks about these things, then if people don’t understand the problem is theirs. But there are some people who think ‘to change sex is a sin, we should remain as God made us, we cannot make physical changes.’ I think that this is not a sin, so this is a natural thing. God made us like this and put us to the test because if it was a sin, why would God put a woman in our body, in my body? If this was a sin, then I would not be like this. If I am like this there is a reason. For this thing, if God made me like this there is a reason. For this I am like this and I do not think this is a sin.

#### 3.2.3. Individuation and the Synthesis of Social Identities

Finally, the third theme highlighted the importance that participants placed on recognizing and defining themselves in gender categories that differed from those of the West. One participant used the Arabic term *aber* to refer to transgender people who were masculine or non-binary. Other participants rejected the transgender label in favor of an identification limited to perceived gender, which (in these cases) concerned the category of “woman” [43].

Transgender people from non-Western cultures should not be expected to use Western terminology to define their gender identity, as such terminology is derived from Anglo-Saxon and North American culture. White privilege blindness frequently leads White and Western people (and researchers) to assume that non-Western cultures use the same Western categories to describe gender identities and sexual orientations—or, worse, to dismiss non-Western experiences and categories related to non-conforming gender identities and sexual orientations altogether. In the present study, participants preferred to use terms from their culture of origin. For example, Ayoub introduced some Arab terms:

Aber, which means ‘trans’ and is used in Arab activism… obviously in dialect there are also other words, maybe they are ugly words. Their ancient origin does not have a beautiful meaning…. Aber is a plural verb, a plural word which is used not only to define transgender males but also non-binary people.

Use of the term “trans” (or “transgender”) has not always been welcome. Accordingly, participants often emphasized their gender identity (in this case, female), rather than their social or medical-surgical background. As Maria stated:

I feel like a woman. I really feel like a woman. That’s why I put it so big because I really feel like a woman, not a trans woman. If I felt like a trans woman, I wouldn’t have the surgery, and I would have stayed like this as a real trans woman. Do you understand? I submitted to the intervention because I really feel like a woman.

In Maria’s case, it was important to distinguish between those who, like her, embarked on a surgical process of gender affirmation, and those who, on the other hand, only desired to undergo hormone therapy to modify their body or manage a social transition. It is noteworthy that people like Ayoub, with a non-binary identity and high level of education, searched within their culture of origin for terms deemed more adequate to reflect their gender fluid identify.

In their individuation process, participants conferred a fundamental role to medical-surgical treatments for gender affirmation (e.g., access to hormones and surgery), which represented methods for bringing one’s gender expression closer to one’s gender identity [44,45]. Medical-surgical treatments have long had gender affirming value for many transgender people [46,47,48,49]. Specifically, hormones and surgery are the medical means by which many transgender people achieve psychophysical wellbeing. Until quite recently, the transgender community used these tools outside of the regulated medical context, accumulating significant knowledge of the use of hormones [50].

Maria’s comment about the self-administration of hormones is noteworthy:

I haven’t [taken the medical route] in Cuba. I would like to do it, but in Cuba, there isn’t a medical course like here in Italy, for example. In Italy, an endocrinologist follows you, gives you [an] orientation, and tells you how to do it, the amount. In Cuba it’s … a la massamba [self-administered]. The old trans passed this information, so it is the trans community that passed the information on.

The massamba way means the good way. This expression points to a distant but not too distant past conception of the health of transgender people and their processes of self-determination. The quotation further brings to mind a practice in the Italian trans community that has almost disappeared. As in Cuba, older Italian transgender women—rather than specialized medical centers—previously initiated younger transgender people into the world of hormones and the search for themselves through them. Indeed, many transgender refugees are still pleasantly surprised to discover specialized health centers for their medical-surgical needs.

## 4. Discussion

The present research used a qualitative instrument to explore transgender refugee experiences of life, migration, and intersectionality, identifying preliminary themes meriting further exploration, given the small number of participants.

Participants’ primary motivation for migration was the minority stress that they experienced in their country of origin [51]. Italy, the host country, was sometimes perceived as a safer environment for accessing gender affirmative medical treatments and expressing one’s gender identity and expression [12,15]. However, some participants reported negative experiences with the international reception and protection services in Italy, highlighting that many transgender asylum seekers risk secondary victimization through the inquisitorial evaluation system that requires adherence to gender models that may not align with their own experiences [14,18,19].

Furthermore, due to their intersecting ethnic and gender identities, some participants reported a sense of isolation from both their communities of ethnic and religious origin and the Italian LGBTQI + community, suffering further negative consequences for mental health [9,16]. However, participants also reported several religious coping strategies that helped them to redefine their religious values and positively combine these with their gender identity [40,41,52]. Participants’ individuation proceeded through their synthesis of social identities and their medical-surgical treatments for gender affirmation [46,47].

Transgender refugees have an awareness of their gender identity that precedes their migration journey. Often from childhood, they set out to understand who they are, exploring their gender identity. Experiences of gender variance have existed for centuries—if not millennia—as recorded in mythology and narratives across cultures. Over time, concepts of sex, gender, sexual orientation, and gender roles have become more complex and differentiated. The sex assigned at one’s birth may differ from one’s gender, which is based on one’s perception of body and gender. Gender roles describe collective behavior and ways of being associated with particular genders, and sexual orientation refers to the object of sexual and romantic attraction. The variety of experiences of gender affirmation and transition inform us that a new way of viewing sex and gender is emerging [53].

The journey of gender affirmation, which may include medical-surgical treatments, is understood as an assisted process through which one moves from an unsatisfactory to a more satisfying life state. The optimal endpoint need not be within a cis/heteronormative framework [2,54]. In achieving psychological wellbeing and quality of life, many transgender and non-binary people find hormone therapies and gender affirming surgery essential. Indeed, through these medical-surgical treatments, many transgender people achieve wellbeing and fulfillment and a more positive identity [46,47,48,49].

As the present study demonstrates and the literature notes [11,12], many transgender refugees are subjected to minority stress early on in their life. Their motivation to migrate is often located in early victimization in the family, and life-long experiences of stigma and discrimination. For some, such minority stress may be experienced in their country of origin within an additionally distressing environment of armed conflict. In most cases, discrimination and violence in the country of origin prevent transgender refugees from openly living their gender identity and limit their access to transgender health services. This, in addition to experiences of stigma, may motivate their risky journey to a new country.

### 4.1. Research and Clinical Implications

The interview protocol applied in the present study was effective for gathering the life stories of transgender refugees. The different sections and questions guided participants to reflect on their development trajectories, migratory motivations, and experiences at the intersection of different minority identities. Social operators may use the interview protocol to collect information required by public authorities to support the claims of transgender asylum seekers. Furthermore, the simplicity of the interview structure enables it to be easily applied, following training, by social operators, social workers, and legal operators.

From a clinical perspective, the interview protocol may be helpful in assessing transgender people with an immigrant and non-Western cultural background. The different sections of the interview may enable practitioners to gradually explore the life experiences and individuation processes of transgender people—including those with trauma and minority stress resulting from both their migration journey and multiple minority identities. Specifically, the first section of the interview protocol allows practitioners to obtain information about the processes involved in the transgender person’s identity construction and their experiences of intersecting social identities perceived as significant for the self [33].

The second section facilitates a deeper exploration of some psychological aspects related to the pre-and post-migration situation, including migratory motivations, challenges, and stressors, both in the country of origin and the host country, as well as coping strategies for managing these challenges. Overall, the Intersection-T is an effective tool for preliminary psychological sessions, as it is capable of generating an overview of a person’s meaningful life experiences, challenges, and resources.

### 4.2. Limitations

The present study presents some limitations. First, the small sample size and the heterogeneity of participants’ cultural backgrounds do not allow for generalization of the research findings. Indeed, we prioritized the “refugee” category in the inclusion criteria, but could not collect data from a specific ethnic group. We acknowledge that experiences related to migration may significantly vary according to the cultural context of origin, and our intention was not to make such cross-cultural differences invisible. However, the study’s main aim was to explore transgender refugee experiences of life before, during, and after migration, and experiences of intersectionality once arrived in Italy, rather than to report experiences from a single ethnic group. Moreover, most participants were trans women, except for one transmasculine non-binary person. As a result, the findings of this study should be considered as primarily representative of transgender women’s experiences of forced migration. Nevertheless, it is also important to consider that, to date, the forced migration of the transgender population mainly affected transgender women, also because they are more likely involved in human trafficking. We hope that future research will more accurately address such limitations through, for instance, the recruitment of transgender refugees from a specific geographic and cultural context, as well as a more balanced involvement of participants in terms of gender.

Second, although one of the primary aims of the study was to capture experiences at the intersection of multiple marginalized identities, such experiences scarcely emerged from our respondents’ transcripts. This may be due to categorical differences between Western and non-Western countries in describing social identities that shape the self. Despite this limitation, we tried to embrace an intersectional perspective across all research steps, from data collection to data analysis, in order to give value to participants’ multiple experiences of stress and resilience related to their ethnic and gender minority identities.

## 5. Conclusions

The current study might be considered a qualitative exploration into the life experiences and intersectionality experienced by transgender refugees in Italy. The study showed that transgender refugees experience different sources of minority stress, both in their country of origin, and in their host country. For the present participants, the main push factors to migration were traumatic experiences and an inability to openly live their gender identity in their country of origin [12,15,51]. Due to the intersection of multiple minority identities, transgender refugees are at risk of experiencing increased isolation, discrimination, and victimization in their host country. Such stressful experiences may even result from interactions with asylum services, due to a lack of training, racial prejudice, and transphobia among service providers [14,18,19].

However, in this study, participants demonstrated a positive individuation process, linked to gender affirmation treatments and the use of coping strategies aimed at successfully combining meaningful social identities (i.e., those pertaining to religion and gender) [40,41,46,47,52]. The findings suggest that national authorities and policymakers should incorporate an intersectional perspective into social policies to account for the needs of transgender refugees (e.g., training on LGBTQI+ issues for service providers). Transgender refugees continue to face discrimination, violence, and difficulties accessing health and social services. We are aware that much work remains to be done regarding the Italian context, and we are motivated to continue our efforts to promote transgender refugees’ wellbeing, inclusion, and positive identity development.

## Figures and Tables

**Table 1:** Participants’ characteristics.

Pseudonym	Age	Gender Identity	Country of Origin	Year of Arrival in Italy	Religion	Education Degree	Socio-Economic Status	Employment Status
Ayoub	29	Non-binary	Libya	2016	Buddhist	Bachelor	Low	Employed
Brenda	34	(Trans)woman	Cuba	2016	Pentecostal	High school	Low	Unemployed
Maria	47	(Trans)woman	Brazil	2003	Catholic	Middle school	Very low	Unemployed
Amina	26	(Trans)woman	Pakistan	2016	Islamic	Middle school	Very low	Unemployed
Serra	28	(Trans)woman	Turkey	2018	Protestant	Bachelor	Very low	Unemployed

**Table 2:** Thematic structure and representative quotations.

Theme	Sub-Theme	Number of Quotations	Representative Quotation
Pre- and post-migration minority stress and transphobia	Country of origin	Ayoub: 8; Maria: 8; Brenda: 5; Amina: 4; Serra: 3	*From my father* […] *it was verbal violence, torture, I experienced domestic torture from my father. I have experienced domestic torture by my father and also my brothers, and they have always tried [to “correct” my gender identity] since I was a child because I was very much towards the male gender. They have tried in every way to feminize my body and my behavior and let’s say the… that violence, I call it violence.* (Ayoub)
	Migration journey	Ayoub: 0; Maria: 1; Brenda: 1; Amina: 2; Serra: 0	*During the trip, I slept in the forests. I didn’t eat, I had a fever, I couldn’t walk, I couldn’t climb the mountains, my knee hurt. So, everything is not just one thing, the whole trip was difficult, but I didn’t lose my courage, and I wanted to go to a country where I could be free.* (Amina)
	Italy (host country)	Ayoub: 5; Maria: 4; Brenda: 4; Amina: 1; Serra: 1	*The hardest thing when I was alone, I missed everything, there was no one near me who could help me.* (Brenda) *Ah ok you used to be a woman, so people like you exist too. Why did you change your mind when you arrived in Italy?’ […] Then I felt anger, I felt hurt, I felt maybe the first time that I feel equal to an Italian trans citizen because there is all trans people even if Italian citizens–the same things and… basically I was angry.* (Ayoub)
Religion as a protective factor for gender affirmation	Affirmative religious communities	Ayoub: 0; Maria: 1; Brenda: 0; Amina: 0; Serra: 2	*Actually I didn’t have a bad experience because I was lucky that the church I was attending and the leader of the church were saying that God welcomes everyone as we are and the same God changes you and you have to reshape yourself in God’s way. That’s why we had this problem.* (Maria) *I chose Christianity but protestant, not Armenian or catholic church, because the evangelist protestant church is more open for LGBT people, this was important for me, to be more accepted, to be more welcome.* (Serra)
	Positive personal synthesis	Ayoub: 4; Maria: 2; Brenda: 0; Amina: 3; Serra: 1	*Yes, in sura, and it is written, it talks about people, God said I can do what I want, I can give him a son as a daughter, and a daughter as a son. These mean he is talking about transgender people. A son is born becomes a daughter. So he talks about these things, then if people don’t understand the problem is theirs.* […] *If this was a sin, then I would not be like this. If I am like this there is a reason. For this thing, if God made me like this there is a reason. For this I am like this and I do not think this is a sin.* (Amina)
Individuation and the synthesis of social identities	Own gender categories	Ayoub: 8; Maria: 3; Brenda: 2; Amina: 9; Serra: 3	*Aber, which means ‘trans’ and is used in Arab activism… obviously in dialect there are also other words, maybe they are ugly words. Their ancient origin does not have a beautiful meaning…. Aber is a plural verb, a plural word which is used not only to define transgender males but also non-binary people.* (Ayoub) *I have always used either trans or travestì to define myself.* (Brenda)
	Medical process of gender affirmation	Ayoub: 3; Maria: 5; Brenda: 2; Amina: 2; Serra: 3	*Hormones are so important because it helps a male body a lot to turn into a female one, it is very important to use these kinds of hormones because it helps so much with the transition.* (Brenda) *I haven’t [taken the medical route] in Cuba. I would like to do it, but in Cuba, there isn’t a medical course like here in Italy, for example. In Italy, an endocrinologist follows you, gives you [an] orientation, and tells you how to do it.* (Maria)

## Data Availability

Anonymized data will be made available upon reasonable request to the corresponding author.
